# Molecular and computational analysis of 45 samples with a serologic weak D phenotype detected among 132,479 blood donors in northeast China

**DOI:** 10.1186/s12967-019-02134-9

**Published:** 2019-11-27

**Authors:** Xu Zhang, Guiji Li, Zhuren Zhou, Chaopeng Shao, Xuying Huang, Lichun Li, Xiaofeng Li, Ying Liu, Hua Fan, Jianping Li

**Affiliations:** 1 Institute of Transfusion Medicine, Liaoning Blood Center, Shenyang, Liaoning China; 2 Key Laboratory of Blood Safety Research of Liaoning Province, Shenyang, Liaoning China; 3 Department of Hematology, The Forth Affiliated Hospital of China Medical University, Shenyang, Liaoning China; 4 Department of Transfusion, the Second People’s Hospital of Shenzhen, Shenzhen, China; 5 Institute of Transfusion Medicine, Harbin Blood Center, Harbin, Heilongjiang China; 6 Department of Pharmacology, School of Pharmacy, China Medical University, Shenyang, Liaoning China

**Keywords:** *RHD* variant, Serological weak D phenotype, Molecular and computational analysis, Weak D, Partial D, DEL

## Abstract

**Background:**

RH1 is one of the most clinically important blood group antigens in the field of transfusion and in the prevention of fetal incompatibility. The molecular analysis and characterization of serologic weak D phenotypes is essential to ensuring transfusion safety.

**Methods:**

Blood samples from a northeastern Chinese population were randomly screened for a serologic weak D phenotype. The nucleotide sequences of all 10 exons, adjacent flanking intronic regions, and partial 5′ and 3′ untranslated regions (UTRs) were detected for *RHD* genes. Predicted deleterious structural changes in missense mutations of serologicl weak D phenotypes were analyzed using SIFT, PROVEAN and PolyPhen2 software. The protein structure of serologic weak D phenotypes was predicted using Swiss-PdbViewer 4.0.1.

**Results:**

A serologic weak D phenotype was found in 45 individuals (0.03%) among 132,479 blood donors. Seventeen distinct *RHD* mutation alleles were detected, with 11 weak D, four partial D and two DEL alleles. Further analyses resulted in the identification of two novel alleles (*RHD weak D 1102A* and *399C*). The prediction of a three-dimensional structure showed that the protein conformation was disrupted in 16 serologic weak D phenotypes.

**Conclusions:**

Two novel and 15 rare *RHD* alleles were identified. *Weak D type 15, DVI Type 3*, and *RHD1227A* were the most prevalent D variant alleles in a northeastern Chinese population. Although the frequencies of the D variant alleles presented herein were low, their phenotypic and genotypic descriptions add to the repertoire of reported *RHD* alleles. Bioinformatics analysis on RhD protein can give us more interpretation of missense variants of *RHD* gene.

## Background

The number of blood group antigens currently recognized by the International Society of Blood Transfusion is 360, and 322 of them are clustered within 36 blood group systems [1]. The Rh blood group system is the most complex among all blood group systems [2]. The D (RH1) antigen is the most immunogenic and clinically significant antigen, which directly affects the hemolytic transfusion reaction and hemolytic diseases of fetuses and newborns [2, 3]. Besides D-positive and D-negative, RhD blood groups have multiple variants, including weak D, partial D and DEL phenotype [4]. A Working Group of the American Association of Blood Banks and College of American Pathologists published a proposal to use the term “serologic weak D phenotype” to distinguish the results of serological weak D testing using anti-human globulin with those of weak D genotyping based on molecular methods [5, 6].

The genetic alterations of *RHD* alleles differentially influence the RhD protein expression level and the number of RhD epitopes [7]. Weak D and most DEL have all D epitopes; partial D lack one or more D epitopes [3]. Anti-D production in RhD negative recipients transfused with blood from weak D, particle D and DEL donors has been reported [8–11]. Therefore, it is of great significance to accurately determine a serologic weak D phenotype. Nearly all serologic weak D phenotypes can be traced back to changes at the DNA level, including nonsense mutations, missense and synonymous mutations, frame shifts, unequal exchange, gene exchange and gene deletion, as well as others. This enables us to study the molecular mechanism of a serologic weak D phenotype at the gene level, and to determine the type of serologic weak D phenotype.

To date, more than 460 *RHD* alleles have been registered and nominated [12–14]. Serologic weak D phenotypes are often found in the blood samples of blood donors and patients, and molecular studies have been mainly conducted in Caucasian and African populations [15–23]. Corresponding research on the diversity of serologic weak D phenotypes has been reported in southern [24–28], but rarely in northeastern populations in China. Herein, we tested samples from a cohort of 132,479 blood donors in northeastern China for the serologic weak D phenotype. We subsequently sequenced the *RHD* gene of the 45 samples identified in this study as showing a serologic weak D phenotype. We built and optimized three-dimensional (3D) models of serologic weak D phenotypes identified in this study to explore their effect on RhD protein structure. Bioinformatics tools were employed to provide computational predictions on the RhD protein structure of serologic weak D phenotypes and enhance our understanding of how mutations affect phenotypes at the same time.

## Materials and methods

### Study participants

All 132,479 samples were collected from blood donors at the Blood Center of Liaoning Province, which is located in northeastern China, over a 5-year period (January 2012 to December 2016). Some donors may have donated repeatedly, which is common in similar large studies of the past and is known not to affect the statistics and conclusions. The study was approved by the Ethics Committee of the Liaoning Blood Center, Liaoning, China.

### Serological studies

The D antigen was serologically determined using a monoclonal anti-D reagent (IgM, Clone BS226, Bio-Rad Medical Diagnostics GmbH Industriestrabe, Germany) using a microplate test protocol and a fully automated blood grouping instrument (Hemo-Type automatic blood group analyzer; GSG Robotix, Milan, Italy). For the microplate test protocol and testing in a fully automatic blood grouping instrument, 6 μL of whole blood from a sample tube was added to 333 μL of 0.9% saline in a test tube and mixed. An erythrocyte suspension (35 μL) was absorbed to a micropore and mixed with 25 μL of anti-D reagent in accordance with the manufacturer’s instructions.

We retested all samples showing negative or equivocal agglutination by tube method with three commercially available anti-D reagents (1-IgM/IgG blend, Clone D175-2 and D415 1E4, Dominion Biologicals Ltd., Dartmouth, Nova Scotia, Canada; 2-IgM/IgG blend, Clone P3X61, P3X21223B10, P3X290 and P3X35, Diagast Ltd, Loos, France; 3-IgM/IgG blend, Clone TH-28 and MS-26, Merck Millipore Ltd, Livingston, UK). An indirect antiglobulin test (IAT) was performed in the case of negative reactions. RhD-negative phenotyping was performed for all samples by hemagglutination using two techniques (microtiter plate and tube); samples were not identified by serologic adsorption–elution techniques. We performed an antibody screening test for blood group alloantibodies on all D-negative and serologic weak D phenotype samples using a gel method (DiaMed-ID, microtyping system, DiaMed China Limited, Hong Kong, China). Routine Rh typing for C, c, E, and e antigens was performed by the tube method with commercial monoclonal immunoglobulin (IgM) reagents (anti-C, Clone MS-24; anti-c, Clone MS-33; anti-E, MS-80 + 258; and anti-e, Clone MS-16 + 21 + 63, Shanghai Hemo-Pharmaceutical & Biological, Inc., Shanghai, China).

### Molecular analysis of genomic DNA

Genomic DNA was extracted from a 0.2 mL blood sample using a DNA whole blood isolation kit (Tiangen Biotech, Beijing, China) in accordance with the manufacturer’s instructions. The *RHD* gene was sequenced in all serologic weak D phenotypes and 117 D antigen negative samples by IAT as previously described [24]. The nucleotide sequences of all 10 exons as well as adjacent flanking intronic regions, including partial 5′ and 3′ untranslated regions (UTRs), were determined (Table 1). Genomic DNA (50 to 100 ng) was used in a 25 μL reaction mix containing 200 mM dNTPs, 0.1 mM of each specific primer, 1.5 mM MgCl_2_, 1× PCR buffer, and 1 unit of GoTaq polymerase (Promega, Madison, WI, USA), supplemented with ddH_2_O. The following PCR program was used: 5 min of denaturation at 95 °C, 35 cycles of 30 s at 94 °C, 30 s at 62 °C (exons 1, 3, 4, 6–10), 30 s at 58 °C (exons 2, 5), and 1 min at 72 °C, followed by a final 10-min extension at 72 °C. The PCR procedure was carried out in a PE-9700 thermal cycler (Applied Biosystems, Foster City, CA, USA). Sequencing data were analyzed with FinchTV software (Geospiza Inc., Seattle, WA, USA) and all results compared to a NCBI Reference Sequence (RefSeq) database number NG_007494.1. The amino acid alignment of RhD was analyzed by CLUSTAL X (version 2.1) and the amino acid sequences used in the analysis were obtained from a protein database (https://www.ncbi.nlm.nih.gov/protein). *RHD* zygosity was determined on all sequencing samples by the presence or absence of a hybrid Rhesus box as described [29].

**Table 1:** Primers for *RHD* gene amplification and sequencing

Primer denotation	Sequence (5′ to 3′)	Specificity	GenBank accession number	Location	Product size (bp)
E1-s(= E1-seq)	TCCATAGAGAGGCCAGCACAA	D	AJ252314	5′UTR − 152 to − 132	340
E1-a	GCTATTTGCTCCTGTGACCACTT	D	Z97363	+40 to +18	
E2-s	TGACGAGTGAAACTCTATCTCGAT	D	U66341	-1060 to -1037	1602
E2-a	GGCATGTCTATTTCTCTCTGTCTAAT	D/CE	U66341, AB035189	+355 to +330	
E2-seq	CCTGGATTCCTTGTGATACACG	D/CE	U66341, U66340	+227 to +206	
E3-s	GTCGTCCTGGCTCTCCCTCTCT	D	AB035190	− 29 to − 8	219
E3-a	CTTTTCTCCCAGGTCCCTCCT	D/CE	AB035192, AB035191	+39 to +19	
E3-seq	GGTCCCTCCTCCCAGCAC	D/CE	AB035192, AB035191	+28 to +11	
E4-s	GCCGACACTCACTGCTCTTAC	D/CE	U77079, U77078	− 36 to − 16	378
E4-a	TGAACCTGCTCTGTGAAGTGC	D	Y10605	+194 to +174	
E4-seq	GGGAGATTTTTTCAGCCAG	D/CE	Y10605, Y10604	+82 to +64	
E5-s	TACCTTTGAATTAAGCACTTCACAG	D	Y10605	− 267 to − 243	1458
E5-a	TTATTGGCTACTTGGTGCC	D/CE	Z97334, AB035197	+1024 to +1006	
E5-seq	AGACCTTTGGAGCAGGAGTG	D/CE	Y10605, Y10604	− 53 to − 34	
E6-s(= E6-seq)	CAGGGTTGCCTTGTTCCCA	D/CE	Z97334, Z97333	− 95 to − 97	274
E6-a	CTTCAGCCAAAGCAGAGGAGG	D	Z97334	+41 to +21	
E7-s	TGCCCATCCCCCTTTGGTGGCC	D	Z97334	− 106 to − 85	411
E7-a	CCAAGGTAGGGGCTGGACAG	D	AB035194	+171 to +152	
E7-seq	GTCTCACCTGCCAATCTGCT	D/CE	Z97334, Z97333	− 41 to − 22	
E8-s	GGTCAGGAGTTCGAGATCAC	D	AB035194	− 593 to − 574	770
E8-a(= E8-seq)	GATGGGGCACATAGACATCC	D/CE	AB035196	+97 to +78	
E9-s(= E9-seq)	GGTCCAGGAATGACAGGGCT	D	AB035196	− 162 to − 143	530
E9-a	CGCTGAGGACTGCAGATAGG	D	AB035185	+294 to +275	
E10-s	CAAGAGATCAAGCCAAAATCAGT	D/CE	AB035185, AB035184	− 67 to − 45	381
E10-a	AGCTTACTGGATGACCACCA	D	X63097	+290 to +271	
E10-seq	CAGTCTGTTGTTTACCAGATGTTGTTAT	D	X63097	3′UTR +261 to +234	

Primers cited from [24]

*s* sense primer, *a* antisense primer, *seq* sequencing primer

### Statistical analysis

Allele frequencies were calculated from corresponding genotype counts. According to Hardy–Weinberg equilibrium, genotype frequency of D negative homozygote is equal to the square of the D negative allele frequency, and genotype frequency of the heterozygote (D variant and D negative) is equal to twice the product of the two allele frequencies. Allele frequencies for each molecular background of serologic weak D and D negative phenotypes were calculated.

### Computational modeling of RhD protein and amino acid substitutions

The 3D structure of the RhD protein was visualized using Swiss-Pdb Viewer 4.1.0 (https://spdbv.vital-it.ch/), which was used to generate models of the selected protein structure for the corresponding amino acid substitutions [30, 31]. Sorting Intolerant From Tolerant (SIFT) [32], Polymorphism Phenotyping algorithmV2 (PolyPhen-2) [33] and Protein Variation Effect Analyzer (PROVEAN) [34] software were used to predict the impact of amino acid substitutions on RhD protein structure.

## Results

### Serological studies

Using routine methods, we screened 132,479 blood donors for D antigen, 131,939 of whom were found to be D + (99.592%), 495 were D − (0.374%), and 45 (0.034%) had a serologic weak D phenotype [5]. They were sorted based on the anti-D agglutination strength using the two routine techniques, and were also tested with three monoclonal anti-D reagents. No blood group alloantibody was detected in the plasmas of 45 serologic weak D phenotype samples.

### Molecular characterization of D variants

We determined the *RHD* sequence of the coding region, adjacent flanking intronic regions, and the partial 5′ and 3′ UTRs in all 45 serologic weak D phenotype samples and detected 17 distinct alleles (Table 2). Twenty-nine individuals were weak D types representing 11 distinct alleles. Twelve individuals were partial D phenotypes representing four distinct alleles. Four individuals were DEL phenotypes representing two distinct alleles. Two novel single-nucleotide missense mutations (c.399G > C and c.1102G > A) were identified in three individuals. The DNA sequences of two novel alleles have been submitted to the GenBank Database (accession numbers KM282169 and KM282170, respectively). Six individuals carried complex alleles: one individual carried *weak D 101G* (c.101A > G) and *weak D type15* (c.845 G > A) alleles, while another five individuals had a weak D (c.399G > C, c.845 G > A, c.763G > C, c.779A > G, c.1212C > A, respectively) and *RHD1227A* allele (c.1227G > A). One each of *weak D type 72* (c.1212C > A), *weak D type 18* (c.19C > T), *weak D type 25* (c.341G > A), *weak D type 31* (c.17C > T) and *weak D type 54* (c.365C > T) were identified.

**Table 2:** *RHD* alleles found among donors with a serologic weak D phenotype

Sample number	*RHD* allele	Occurrence frequency (%)	Phenotype annotation	Nucleotide change	Amino acid substitution	Haplotype	Hybrid Rhesus box results	ISBT terminology	References
1–16	Weak D type 15	16 (35.6)	Weak D	c.845G > A	p.G282D	cDE/CDE/CDe	*RHD*+*/RHD*−	RHD*15	[35]
17	Weak D type 15 *RHD* 1227A	1 (2.2)	Weak D	c.845G > A c.1227G > A	p.G282D p.K409K	CDe	*RHD+/RHD*+	RHD*15 RHD*01EL.01	[35, 36]
18	Weak D type 18	1 (2.2)	Weak D	c.19C > T	p.R7W	CDe	*RHD+/RHD*−	RHD*01 *W.18*	[37]
19	Weak D type 25	1 (2.2)	Weak D	c.341G > A	p.R114Q	CDe	*RHD+/RHD*−	RHD*01 *W.25*	[38]
20	Weak D type 31	1 (2.2)	Weak D	c.17C > T	p.P6L	CDe	*RHD+/RHD*−	RHD*01W.31	[39]
21	Weak D type 54	1 (2.2)	Weak D	c.365C > T	p.S122L	CDe	*RHD+/RHD*−	RHD*01W.54	[38]
22	Weak D type 72	1 (2.2)	Weak D	c.1212C > A	p.D404E	CDe	*RHD+/RHD*−	RHD*01W.72	
23	Weak D type 72 *RHD* 1227A	1 (2.2)	Weak D	c.1212C > A c.1227G > A	p.D404E p.K409K	CDe	*RHD+/RHD*+	RHD*01W.72 RHD*01EL.01	[36]
24	*RHD* weak D 763C *RHD* 1227A	1 (2.2)	Weak D	c.763G > C c.1227G > A	p.G255R p.K409K	CDe	*RHD+/RHD*+	RHD*01EL.01	[36]
25	*RHD* weak D 101G Weak D type 15	1 (2.2)	Weak D	c.101A > G c.845G > A	p.Y34C p.G282D	CDe	*RHD+/RHD*+	RHD*15	[25, 35]
26	*RHD* weak D 399C *RHD* 1227A	1 (2.2)	Weak D	c.399G > C c.1227G > A	p.K133N p.K409K	CDe	*RHD+/RHD*+	RHD*01EL.01	[36]
27	*RHD* weak D 779G *RHD* 1227A	1 (2.2)	Weak D	c.779A > G c.1227G > A	p.H260R p.K409K	CDE	*RHD+/RHD*+	RHD*01EL.01	[25, 36]
28–29	*RHD* weak D 1102A	2 (4.4)	Weak D	c.1102G > A	p.G368R	CDe	*RHD+/RHD*−		
30	weak D type 61	1 (2.2)	DEL	c.28C > T	p.R10W	CDe	*RHD+/RHD*−	RHD*01 *W.61*	[40]
31–33	*RHD* 1227A	3 (6.7)	DEL	c.1227G > A	p.K409K	CDe/CDE	*RHD+/RHD*−	RHD*01EL.01	[36]
34	DFR type2	1 (2.2)	Partial D	*RHD (D1*-*3 CE4 D5*-*10)*		CDe	*RHD+/RHD*−	RHD*17.02	[41]
35–37	D V type 2	3 (6.7)	Partial D	*RHD (D1*-*4CE3*-*5 D6*-*10)*		cDE/CDE	*RHD+/RHD*−	RHD*05.02	[42]
38–44	DVI type 3	7 (15.6)	Partial D	*RHD (D1*-*2*-*CE3*-*6*-*D7*-*10)*		CDe	*RHD+/RHD*−	RHD*06.03	[43]
45	DVI type 4	1 (2.2)	Partial D	*RHD (D1*-*2 CE3*-*5 D6*-*10)*		CDe	*RHD+/RHD*−	RHD*06.04	[44]

*ISBT* International Society of Blood Transfusion

### Calculation of allele frequencies

In our study, phenotype frequencies of serologic weak D and D negative phenotypes were 0.00034 and 0.00374, respectively. According to calculation, the D negative allele frequency was 0.06113, and frequency of a single D variant allele observation was 0.00005. In 117 D negative samples, 66.67% of the genetically tested RhD negatives seemed to be homozygous for the *RHD* deletion, and 33.33% had a deletion of *RHD* plus a variant *RHD* or a *RHD*-*CE*-*D* hybrid gene. Cumulative *RHD 1227A* allele frequency among weak D and D negative phenotypes was 0.00853. Expected homozygous deletion of *RHD* had a genotype frequency of 0.00244. Allele frequencies of serologic weak D phenotypes and D negative alleles were calculated as shown in Tables 3 and 4. Raw data for calculation of serologic weak D and RhD negative allele frequencies are shown in Additional file 1: Table S1.

**Table 3:** Allele frequencies of serologic weak D phenotype alleles

Genetically tested variants	Sample number	*RHD neg*	Weak D15	Weak D 18	Weak D 25	Weak D31	Weak D54	Weak D72	Weak D763C	Weak D101G
Weak D type 15/*RHD neg*	16	16	16							
Weak D type 15*/RHD* 1227A	1		1							
Weak D type 18/*RHD neg*	1	1		1						
Weak D type 25/*RHD neg*	1	1			1					
Weak D type 31/*RHD neg*	1	1				1				
Weak D type 54/*RHD neg*	1	1					1			
Weak D type 72/*RHD neg*	1	1						1		
Weak D type 72*/RHD* 1227A	1							1		
Weak D 763C/*RHD* 1227A	1								1	
Weak D101G/weak D type 15	1		1							1
Weak D 399C*/RHD* 1227A	1									
Weak D 779G/*RHD* 1227A	1									
Weak D 1102A/*RHD neg*	2	2								
Weak D type 61/*RHD neg*	1	1								
*RHD* 1227A/*RHD neg*	3	3								
DFR type2/*RHD neg*	1	1								
D V type 2/*RHD neg*	3	3								
DVI type 3/*RHD neg*	7	7								
DVI type 4/*RHD neg*	1	1								
Totals	45	39	18	1	1	1	1	2	1	1
Allele frequency			0.00096	0.00005	0.00005	0.00005	0.00005	0.00011	0.00005	0.00005

**Table 4:** Allele frequencies of RhD negative alleles

Genetically tested RHD negative	Sample number	*RHD neg*	*RHD 1227A*	*RHD 1166delA*	*RHD*-*Ce (2*-*9)*-*D*	*RHD 711delC*
*RHD neg/RHD neg*	78	156				
*RHD neg/RHD 1227A*	24	24	24			
*RHD 1227A/RHD 1227A*	3		6			
*RHD 1227A/RHD 1166delA*	1		1	1		
*RHD neg/RHD*-*Ce (2*-*9)*-*D*	7	7			7	
*RHD*-*Ce (2*-*9)*-*D/RHD*-*Ce (2*-*9)*-*D*	2				4	
*RHD neg/RHD 711delC*	2	2				2
Totals	117	189	31	1	11	2
Allele frequency		0.04937	0.00810	0.00026	0.00287	0.00052

### Predicted effect of nonsynonymous substitutions

**Fig. 1:**
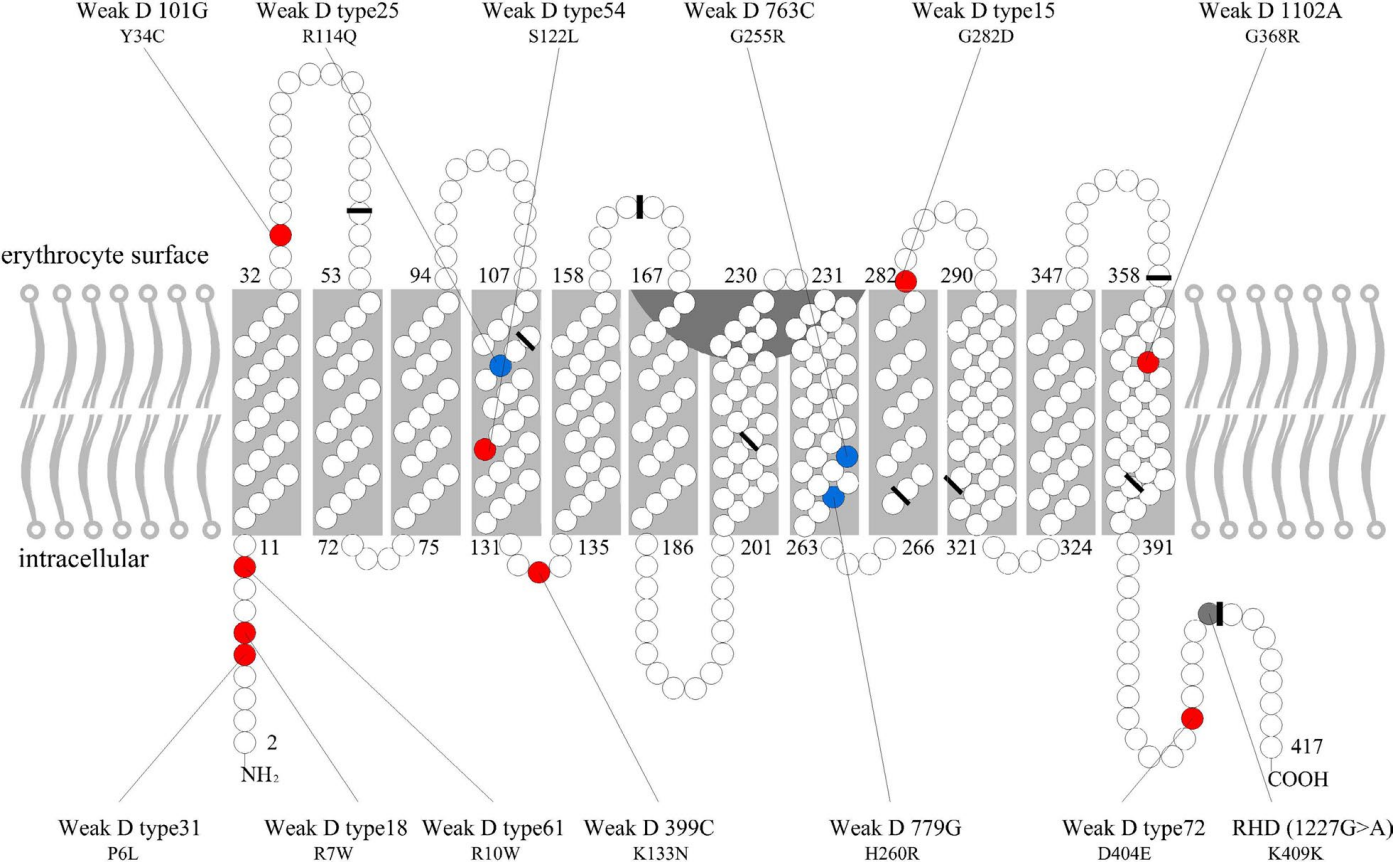
Positions of single amino acid substitution of the RhD protein (adapted from Flegel [4] and Srivastava [45]). There are 417 amino acids in the RhD protein, shown here as circles. The mature protein in the cell membrane lacks the first amino acid. The nine exon boundaries in the *RHD* cDNA as reflected in the amino acid sequence are labeled as black bars. All detected amino acid substitutions encoding D variant alleles are labeled as colored circles. A synonymous single nucleotide polymorphism (SNP) caused no amino acid change (gray). The other SNPs are nonsynonymous and cause amino acid changes that are predicted to affect the RhD protein structure (red) or to be neutral (blue)

SIFT, PolyPhen-2, and PROVEAN bioinformatic software programs were used to predict deleterious structural changes induced by nonsynonymous p.P6L, p.R7W, p.R10W, p.Y34C, p.R114Q, p.S122L, p.K133N, p.G255R, H260R, p.G282D, p.G368R, and p.D404E substitutions (Additional file 2: Table S2). The 12 nonsynonymous substitutions were distributed along the whole length of the *RHD* coding sequence without any apparent clustering (Fig. 1) [4, 45].

### Bioinformatics analysis of RhD protein structure model

**Fig. 2:**
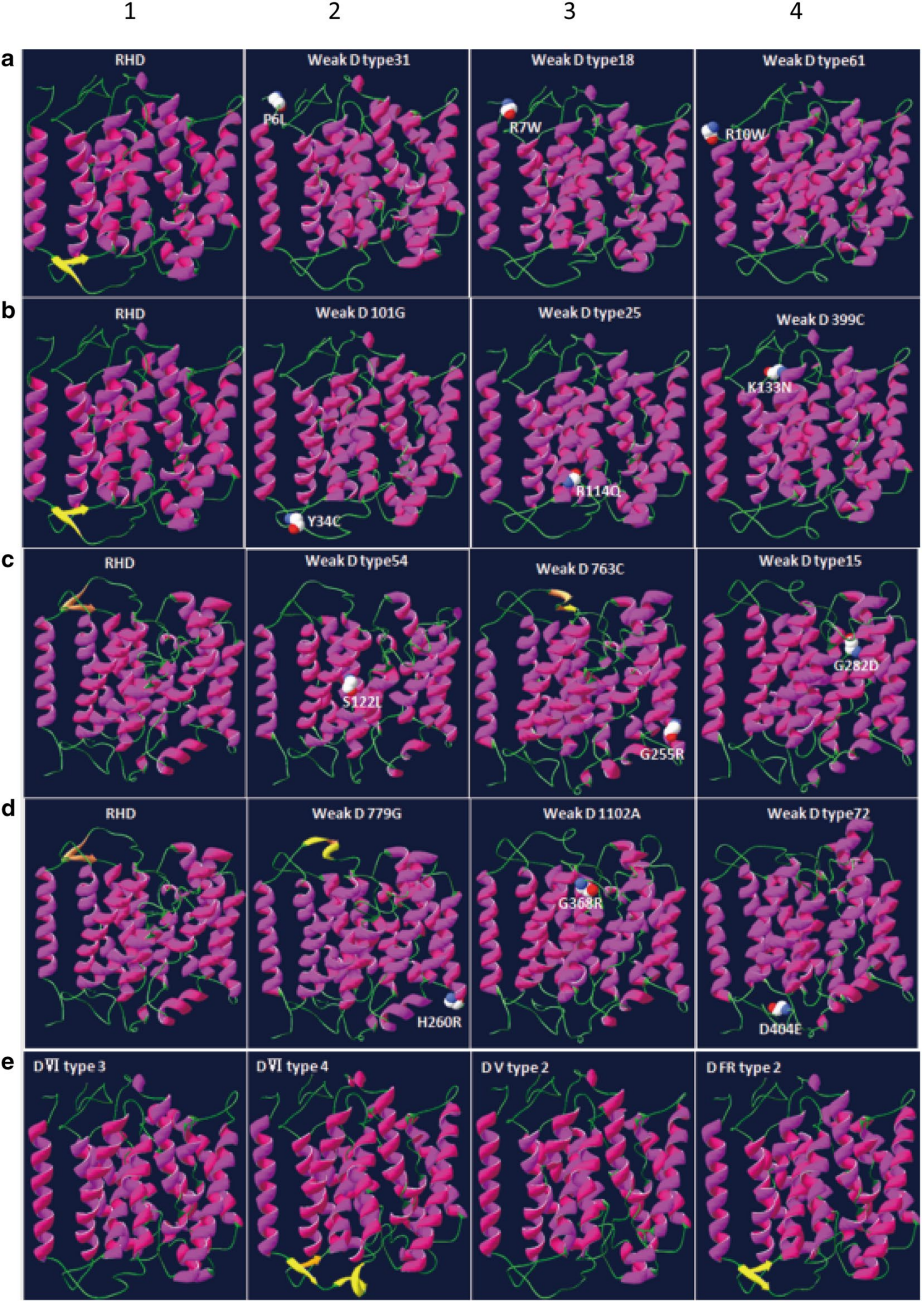
Comparison of the tertiary structure of modeled wild-type and mutant RhD proteins. *RHD* and D variant tertiary structures were modeled using Deep View-Swiss-Pdb Viewer 4.0.1. Helices are shown as deep pink and rose pink ribbons, sheets as yellow and orange, and coils as green. **a1**, **b1**, **c1**, and **d1** are wild-type RhD protein. The weak D proteins of weak D type 31 (**a2**), weak D type 18 (**a3**), weak D type 61 (**a4**), weak D 101G (**b2**), weak D type 25 (**b3**), weak D 399C (**b4**), weak D type 54 (**c2**), weak D type 763C (**c3**) , weak D type15 (**c4**), weak D779G (**d2**), weak D 1102A (**d3**), and weak D type 72 (**d4**) compared with the left wild-type RhD proteins, respectively. The partial D proteins of DVI type 3 (**e1**), DVI type 4 (**e2**), D V type 2 (**e3**) and DFR type2 (**e4**) compared with the wild-type RhD protein (**a1**), respectively. The mutant weak D proteins (p.P6L, p.R7W, p.R10W, p.Y34C, p. R114Q, p.K133N, p.S122L, p.G255R, p.G280D, p.H260R, p.G368R and p.D404E) are highlighted

The template of RhD protein homology model was in accordance with the model based on computational hydropathy map [45, 46]. The model comprised 409 amino acids from Ser3 to Pro411 and lacked nine residues (two in the N terminus and six in the C terminus, Additional file 3: Table S3). The RhD 3D protein structures of the wild-type and 16 serologic weak D phenotypes highlighted the change in structure with altered amino acids. A 3D structure analysis of 16 serologic weak D phenotypes predicted amino acid position shifts in intracellular and exofacial loops, and the transmembraneous domain. The 3D structure model also demonstrated that p.P6L, p.R7W, p.R10W, p.Y34C, p.R114Q, p.S122L, p.K133 N, p.G280D, p.G368R and p.D404E mutations led to the disappearance of beta sheets, and position changes due to p.G255R and p.H260R mutations in beta sheets. The 3D structure model of four partial D types displayed the disappearance of beta sheets in *DVI type 3* and *DV type 2*, an increase in beta sheets at amino acids 38–40 and 42–44 in *DVI type 4*, and no change in beta sheets in *DFR type 2* only, with an amino acid position shift in intracellular and exofacial loops, and the transmembraneous domain. The 3D structure model of four partial D types displayed the disappearance of beta sheets in *DVI type 3* and *DV type 2*, an increase in beta sheets at amino acids 38–40 and 42–44 in *DVI type 4*, and no change in beta sheets in *DFR type 2* only, with an amino acid position shift in intracellular and exofacial loops, and the transmembraneous domain (Fig. 2).

## Discussion

In the present study, we investigated the molecular characteristics of serologic weak D phenotypes in a northeastern population in China. The bioinformatics of 17 variant *RHD* alleles for serologic weak D phenotypes, including two novel alleles, were analyzed. The *RHD* allele distribution varies widely between Asian [24–28, 47–49] and European centers [15–21]. Differences in populations and routine serologic screening procedures employed, as well as in the molecular examinations used, may account for such differences to date, highlighting the need for standardization. In this study, *Weak D type 15, DVI Type 3 and DEL (RHD1227A)* were the most prevalent D variant alleles measured in the northeastern Chinese population, which were consistent with those reported in southern Chinese population [24, 25, 27]; however, they were rare in other populations. Therefore, the frequency of distribution of serologic weak D phenotypes varies among populations and ethnic groups.

Our tests detected two mutation types for DEL variants. One type was *RHD1227A* (c.1227G > A), and the other was *weak D type 61* (c.28C > T). *Weak D type 61* was determined on the basis of weak agglutination in the IAT procedure; it was first reported in the Chinese population [40]. The DEL *(RHD1227A*) variant with very low levels of D antigen detectable only by the adsorption-elution method accounts for 10% to 33% of apparent D negative phenotypes in eastern Asia [26, 40, 50]. As estimated, the maximum antigen site density per red cell was 36 and often no more than 22 [51]. In this study, *RHD1227A* was detected eight in 45 serologic weak D phenotypes and 28 in 117 RhD negative individuals by sequencing. Primary and second immunization of RhD negatives by *RHD1227A* blood have been shown to occur [10, 11, 52]. First, as the measure for improvement of transfusion safety in China, RhD negative individuals should be *RHD* genotyped, in order to reduce the number of immunizations of RhD negatives with *RHD1227A* positive blood, not identified by standard serological techniques. Second, testing of “serological weak D phenotype” donors would be of interest among donors, of higher importance in recipients. For blood samples of patients or donors with serological weak D phenotype, some are hard to determine serologically. Patients with several serologic weak D phenotypes (DFR, DV, DVI, and Weak D type 15) reported in this study have been found to develop alloanti-D [3]. Therefore, it is necessary to identify different *RHD* alleles and their frequencies in different populations. More practical investigations of Rh-related transfusion and obstetrics in China and other Asian populations are encouraged.

The 12 nonsynonymous variant mutations were dispersed throughout RhD protein, with no clustering at specific sites. They occurred in the intracellular, exofacial, and transmembraneous red blood cell membrane (Fig. 1). While weak D phenotypes derived mainly from amino acid substitutions in intracellular or transmembrane segments of RHD, partial D is located in extracellular portions of the *RHD* polypeptide [11, 35]. In this study, 12 weak D mutations were found in the intracellular and transmembrane region, except *weak D101G* (c.101 A > G) [25]. The possible reason is that the precise locations of the amino acid residues of RhD protein in the membrane is not yet clear; different models may predict the different locations of some amino acids [3].The substitutions may also affect the tertiary interactions and stabilization of the RhD protein. The prediction of 3D structures showed that the space conformation of the protein was disrupted in 16 serologic weak D phenotypes. These all affect the normal assembly of the tertiary structure, resulting in an activity change of the D antigen. These results indicate that bioinformatics analysis on RhD protein can give us more interpretation of missense variants of *RHD* gene.

The *RHD* gene coding region, splicing sites, partial introns, and 5′ and 3′ UTRs were detected in 45 samples with serologic weak D phenotypes in this study. The mutation sites of the 45 samples were all located in the coding region. At present, most studies on the serologic weak D phenotype are at the DNA level, and relevant available RNA information is not comprehensive. Therefore, the molecular mechanism(s) underlying serologic weak D phenotypes need to be further investigated. In addition, due to the relative scarcity of RhD negative samples in the Chinese population, especially that of serologic weak D phenotype samples, data about the overall characteristics of various ethnic groups in China are still relatively lacking at the present time. Therefore, increased specimen collection is an urgent problem that remains unresolved.

## Conclusions

This study describes two novel and 15 rare *RHD* alleles by variant screening of large doubtful D phenotypes and provides a brief overview of serologic weak D phenotypes with respect to their underlying mutational mechanisms. We also applied bioinformatics analysis to predicted deleterious structural changes of serologic weak D phenotypes. These data extend our knowledge of serologic weak D phenotypes in blood donors and clinical transfusion recipients, which underlies the safety of blood transfusion and which may reduce the risk of anti-D immunization.

## Supplementary information

**Additional file 1: Table S1.** The calculation of allele frequencies for serologic weak D phenotype and RhD negative alleles.

**Additional file 2: Table S2.** Predicted effects on RhD protein function based on SNP missense mutations.

**Additional file 3: Table S3.** Predicted 3D structure of 16 serologic weak D phenotypes with the amino acid position shifts in the intracellular loop, transmembraneous domain and exofacial loop.

## Data Availability

Not applicable.

## Abbreviations

IAT: an indirect antiglobulin test
UTRs: untranslated regions
SNP: single nucleotide polymorphism
3D: three-dimensional
SIFT: Sorting Intolerant From Tolerant
PolyPhen-2: Polymorphism Phenotypingv2
PROVEAN: protein Variation Effect Analyzer
