# Supplementary material for: Molecular and computational analysis of 45 samples with a serologic weak D phenotype detected among 132,479 blood donors in northeast China
Source: J Transl Med. 2019 Nov 27;17:393. doi: 10.1186/s12967-019-02134-9 (PMC6880393; doi:10.1186/s12967-019-02134-9)
Supplement: Supplementary file 1 — Additional file 1: Table S1. The calculation of allele frequencies for serologic weak D phenotype and RhD negative alleles. [file 12967_2019_2134_MOESM1_ESM.doc]

**Table S1 . The calculation of allele frequencies for serologic weak D phenotype and RhD negative alleles**

serological weak D

D pos D neg phenotypes total

131,939 495 45 132,479

phenotype frequency: 0.99592 0.00374 0.00034 1.00000

allele frequency: 0.06113

|  | hemizygous (1 RHD) |  | compound heterozygous  (2 RHDvars) |
| --- | --- | --- | --- |

individuals 39 6

RHD haplotypes 39 12

0,00034 = 2*0,06113*(RHD-frq) +(RHDvar-frq)2

0,00034 = 0,12225*(RHD-frq) +(RHDvar-frq)2

0 = (RHD-frq)^2^ + 0,12225*(RHD-frq) - 0.00034

All (RHDvar-frq)1 =-0.124980426

All (RHDvar-frq)2 =0.002720426

39 plus 12 alleles = 51

single (RHDvar-frq) = 0.0000533

RHD neg halotype : 0.06113

| **Allele frequencies of major serologic weak D phenotype** | | | **Allele frequencies of RhD negative alleles** | | |
| --- | --- | --- | --- | --- | --- |
| Allele | number | Allele frequency | Allele | number | Allele frequency |
| *weak D type 15* | 18 | 0.00096 | *RHD neg* | 189 | 0.04937 |
| *RHD 1227A* | 8 | 0.00043 | *RHD 1227A* | 31 | 0.00810 |
| *DVI type 3* | 7 | 0.00037 | *RHD 1166delA* | 1 | 0.00026 |
| *weak type 72* | 2 | 0.00011 | *RHD-Ce (2-9)-D* | 11 | 0.00287 |
| *weak D 1102A* | 2 | 0.00011 | *RHD 711delC* | 2 | 0.00052 |

For the other weak D allelles whose allele number is one, allele frequency is 0.00005.

*RHD 1227A* allele frequency in 132,479 blood donors: 0.00043+0.00810=0.00853

Expected homozygous RHD neg/RHD neg (deletion type) genotypes: 0.0.4937*0.04937=0.00244

pos=postive; neg=negiative; frq=frequency; var=variant
