# Supplementary material for: Molecular and computational analysis of 45 samples with a serologic weak D phenotype detected among 132,479 blood donors in northeast China
Source: J Transl Med. 2019 Nov 27;17:393. doi: 10.1186/s12967-019-02134-9 (PMC6880393; doi:10.1186/s12967-019-02134-9)
Supplement: Supplementary file 2 — Additional file 2: Table S2. Predicted effects on RhD protein function based on SNP missense mutations. [file 12967_2019_2134_MOESM2_ESM.doc]

| **Table S2. Predicted effect on RhD protein structure based on SNP missense mutations** | | | | | | |
| --- | --- | --- | --- | --- | --- | --- |
| Allele name | Nucleotide change | substitution | SIFT* | PROVEAN† | PolyPhen-2‡ | Prediction§ |
| weak D type 31 | c.17C>T | p.P6L | 0.01 | -4.364 | 0.999 | Deleterious |
| weak D type 18 | c.19C>T | p.R7W | 0.03 | -3.236 | 0.034 | Deleterious |
| weak D type 61 | c.28C>T | p.R10W | 0.00 | --6.475 | 1.000 | Deleterious |
| weak D 101G | c.101A>G | p.Y34C | 0.01 | -6.684 | 1.000 | Deleterious |
| weak D type 25 | c.341G>A | p.R114Q | 0.62 | -0.003 | 0.083 | Neutral |
| weak D type 54 | c.365C>T | p.S122L | 0.10 | -3.575 | 0.903 | Deleterious |
| weak D 399C | c.399G>C | p.K133N | 0.00 | -4.247 | 0.940 | Deleterious |
| weak D 763C | c.763G>C | p.G255R | 0.06 | -0.253 | 0.056 | Neutral |
| weak D 779G | c.779A>G | p.H260R | 0.13 | -4.533 | 0.067 | Neutral |
| weak D type 15 | c.845G>A | p.G282D | 0.01 | -6.078 | 0.975 | Deleterious |
| weak D 1102A | c.1102G>A | p.G368R | 0.00 | -4.649 | 1.000 | Deleterious |
| weak D type 72 | c.1212C>A | p.D404E | 0.00 | -3.074 | 1.000 | Deleterious |

* score ≤0.05 = damaging, >0.05 = tolerated

†score >−2.5 = neutral, ≤−2.5 = deleterious

‡score 0.000–0.452 = benign, 0.453–0.956 = possibly damaging, 0.957–1.000 = probably damaging

§To qualify as deleterious, the mutation had to be predicted as damaging by at least two of the three bioinformatics programs used.

SNP, single nucleotide polymorphisms; SIFT, Sorting Intolerant From Tolerant; PROVEAN, Protein Variation Effect Analyzer; PolyPhen-2, Polymorphism Phenotypingv2
