# Supplementary material for: Molecular and computational analysis of 45 samples with a serologic weak D phenotype detected among 132,479 blood donors in northeast China
Source: J Transl Med. 2019 Nov 27;17:393. doi: 10.1186/s12967-019-02134-9 (PMC6880393; doi:10.1186/s12967-019-02134-9)
Supplement: Supplementary file 3 — Additional file 3: Table S3. Predicted 3D structure of 16 serologic weak D phenotypes with the amino acid position shifts in the intracellular loop, transmembraneous domain and exofacial loop. [file 12967_2019_2134_MOESM3_ESM.doc]

**Table S3. Predicted 3D structure of 16 serologic weak D phenotypes with the amino acid position shifts in the intracellular loop, transmembraneous domain and exofacial loop**

| Allele Location | RHD | weak D type 15 | weak D type18 | weak D type 25 | weak D type 31 | weak D type54 | weak D type72 | weak D 101G | weak D 399C | weak D763C | weak D779G | weak D 1102A | RHD Del 8 | DⅥ type 3 | DⅥ type 4 | DV type 2 | DFR type 2 |
| --- | --- | --- | --- | --- | --- | --- | --- | --- | --- | --- | --- | --- | --- | --- | --- | --- | --- |
| IC1 | 3-11 | 3-9 | 3-9 | 3-9 | 3-9 | 3-9 | 3-9 | 3-9 | 3-9 | 3-9 | 3-9 | 3-9 | 10-12 | 3-9 | 3-9 | 3-9 | 3-9 |
| TM1 | 12-30 | 10-30 | 10-30 | 10-30 | 10-30 | 10-30 | 10-30 | 10-30 | 10-30 | 10-27 | 10-30 | 10-30 | 13-30 | 10-30 | 10-30 | 10-30 | 10-30 |
| EC1 | 31-47 (33-35s） | 31-47 | 31-47 | 31-47 | 31-47 | 31-47 | 31-47 | 31-47 | 31-47 | 28-47(38-40s,43-45s) | 31-47(36-38s,40-42s) | 31-47 | 31-47 | 31-47 | 31-47(33-35s,38-40s42-44s) | 31-47 | 31-47 (34-35s） |
| TM2 | 48-66 | 48-66 | 48-66 | 48-66 | 48-66 | 48-66 | 48-66 | 48-66 | 48-66 | 48-66 | 48-66 | 48-66 | 48-66 | 48-66 | 48-66 | 48-66 | 48-66 |
| IC2 | 67-72 | 67-72 | 67-72 | 67-72 | 67-72 | 67-72 | 67-72 | 67-72 | 67-72 | 67-72 | 67-72 | 67-72 | 67-72 | 67-72 | 67-72 | 67-72 | 67-72 |
| TM3 | 73-98 | 73-98 | 73-97 | 73-98 | 73-98 | 73-98 | 73-98 | 73-98 | 73-97 | 73-98 | 73-97 | 73-97 | 73-98 | 73-97 | 73-97 | 73-98 | 73-97 |
| EC2 | 99-110 (106-108s) | 99-110 | 98-110 | 99-109 | 99-110 | 99-109 | 99-109 | 99-109 | 98-110 | 99-110 | 98-109 | 98-110 | 99-110 | 98-109 | 99-109 (106-108s) | 99-110 | 99-109 (106-108s) |
| TM4 | 111-125 | 111-125 | 111-128 | 110-130 | 111-130 | 110-125 | 110-130 | 110-125 | 111-130 | 111-128 | 110-128 | 111-130 | 111-125 | 110-125 | 110-125 | 111-130 | 110-130 |
| IC3 | 126-135 | 126-135 | 129-135 | 131-135 | 131-135 | 126-135 | 131-135 | 126-135 | 131-135 | 129-135 | 129-135 | 131-135 | 126-135 | 126-135 | 126-135 | 131-135 | 131-135 |
| TM5 | 136-158 | 136-159 | 136-158 | 136-158 | 136-159 | 136-158 | 136-158 | 136-158 | 136-158 | 136-158 | 136-158 | 136-157 | 136-158 | 136-158 | 136-159 | 136-158 | 136-158 |
| EC3 | 159-172 | 160-172 | 159-172 | 159-172 | 160-172 | 159-172 | 159-168 | 159-172 | 159-172 | 159-171 | 159-172 | 158-172 | 159-169 | 159-172 | 160-172 | 159-169 | 159-172 |
| TM6 | 173-186 | 173-186 | 173-186 | 173-186 | 173-186 | 173-186 | 169-186 | 173-186 | 173-186 | 172-186 | 173-186 | 173-186 | 170-186 | 173-186 | 173-186 | 170-186 | 173-186 |
| IC4 | 187-202 | 187-202 | 187-202 | 187-202 | 187-202 | 187-202 | 187-202 | 187-202 | 187-202 | 187-202 | 187-202 | 187-202 | 187-202 | 187-202 | 187-202 | 187-202 | 187-202 |
| TM7 | 203-223 | 203-225 | 203-223 | 203-222 | 203-222 | 203-223 | 203-226 | 203-222 | 203-222 | 203-223 | 203-225 | 203-223 | 203-222 | 203-225 | 203-223 | 203-217 | 203-225 |
| EC4 | 224-230 | 226-230 | 224-230 | 223-230 | 223-230 | 224-230 | 227-230 | 223-230 | 223-230 | 224-230 | 226-230 | 224-230 | 223-230 | 226-230 | 224-230 | 218-230 | 226-230 |
| TM8 | 231-259 | 231-259 | 231-259 | 231-259 | 231-259 | 231-259 | 231-259 | 231-259 | 231-259 | 231-259 | 231-259 | 231-259 | 231-259 | 231-256 | 231-259 | 231-259 | 231-259 |
| IC5 | 260-267 | 260-266 | 260-267 | 260-266 | 260-266 | 260-266 | 260-266 | 260-266 | 260-266 | 260-266 | 260-266 | 260-266 | 260-266 | 257-267 | 260-267 | 260-268 | 260-267 |
| TM9 | 268-287 | 267-285 | 268-279 | 267-279 | 267-285 | 267-285 | 267-287 | 267-283 | 267-285 | 267-285 | 267-285 | 267-287 | 267-280 | 268-283 | 267-285 | 269-281 | 268-283 |
| EC5 | 288-291 | 286-291 | 280-291 | 280-291 | 286-291 | 286-291 | 288-291 | 284-291 | 286-291 | 286-291 | 286-291 | 288-291 | 281-291 | 284-291 | 286-291 | 282-291 | 284-291(287h) |
| TM10 | 292-319 | 292-314 | 292-314 | 292-318 | 292-319 | 292-319 | 292-319 | 292-319 | 292-314 | 292-314 | 292-319 | 292-314 | 292-314 | 292-320 | 292-319 | 292-315 | 292-319 |
| IC6 | 320-331 | 315-331 | 315-332 | 319-331 | 320-332 | 320-331 | 320-332 | 320-332 | 315-331 | 315-331 | 320-328 | 315-331 | 315-327 | 321-333 | 320-331 | 316-333 | 320-331 |
| TM11 | 332-347 | 332-346 | 333-347 | 332-347 | 333-347 | 332-346 | 333-347 | 333-347 | 332-346 | 332-347 | 329-347 | 332-344 | 328-346 | 334-347 | 332-346 | 334-348 | 332-347 |
| EC6 | 348-373 | 347-369 | 348-369 | 348-369 | 348-369 | 347-369 | 348-369 | 348-372 | 347-369 | 348-373 | 348-369 | 345-369 | 347-369 | 348-369 | 347-369 | 349-374 | 348-369 |
| TM12 | 374-388 | 370-388 | 370-388 | 370-388 | 370-388 | 370-388 | 370-388 | 373-388 | 370-388 | 374-388 | 370-388 | 370-388 | 370-388 | 370-388 | 370-388 | 375-389 | 370-388 |
| IC7 | 389-411 | 389-411 | 389-411 | 389-411 | 389-411 | 389-411 | 389-411 | 389-411 | 389-411 | 389-411 | 389-411 | 389-411 | 389-411 | 389-411 | 389-411 | 390-412 | 389-411 |

EC = extracellular loop; IC = intracellular loop; TM = transmembrane domain; s= sheet; h=helix
